# Emergency colon cancer diagnosis in people with mental health conditions: a population-based cohort study in northern Italy

**DOI:** 10.1136/bmjment-2025-301733

**Published:** 2025-07-01

**Authors:** Flavia Pennisi, Carlotta Buzzoni, Federico Gervasi, Antonio Giampiero Russo, Cristina Renzi

**Affiliations:** 1 Vita-Salute San Raffaele University Faculty of Medicine and Surgery, Milan, Italy; 2 Department of Public Health, Experimental and Forensic Medicine Pavia, University of Pavia, Pavia, Lombardia, Italy; 3 Epidemiology Unit, Agenzia di Tutela della Salute della Citta Metropolitan di Milano, Milano, Lombardia, Italy; 4 Research Department of Behavioural Science and Health, University College London, London, UK

**Keywords:** Adult psychiatry, Depression & mood disorders, Personality disorders, Schizophrenia & psychotic disorders, Substance misuse

## Abstract

**Background:**

Individuals with mental health conditions may experience disparity in cancer diagnosis and health outcomes. This study aims to examine diagnostic pathways and mortality in patients with colon cancer with pre-existing mental health conditions.

**Methods:**

A population-based cohort study on colon cancer cases diagnosed in 2014–2020 in the provinces of Milan and Lodi, using linked cancer registration and health data. We examined cancer diagnostic pathways (screening, emergency presentation (EP), inpatient/outpatient visits) and short-term mortality in patients with and without pre-existing mental health conditions, accounting for physical comorbidities and sociodemographic factors. Mental health conditions were systematically categorised into distinct groups according to the International Classification of Diseases, 10th Revision.

**Results:**

Out of 11 429 patients with colon cancer, 16.2% had a pre-existing mental health condition. Individuals with mental health conditions versus those without had a higher risk of cancer diagnosis following EP: 43.8% versus 33.8%, adjusted OR (aOR) 1.32, 95% CI 1.19 to 1.47. EP risk was higher for patients with diagnoses of dementia and related cognitive conditions (aOR 1.69, 95% CI 1.41 to 2.03), substance use/behavioural syndromes/personality-related conditions (aOR 1.92, 95% CI 1.34 to 2.75) and anxiety (aOR 1.44, 95% CI 1.16 to 1.79). The likelihood of screening-detected cancer was lower (4.6% vs 9.1%; aOR 0.78, 95% CI 0.60 to 0.99), especially for dementia and related cognitive conditions (aOR 0.27, 95% CI 0.08 to 0.86). Short-term mortality was higher in patients with cancer with mental health conditions than in those without.

**Conclusion:**

Mental health conditions were associated with a lower likelihood of screening and a higher risk of emergency cancer diagnosis. Tailored strategies are warranted to enhance cancer diagnosis for the non-negligible group of individuals with mental health conditions.

**WHAT IS ALREADY KNOWN ON THIS TOPIC**

- People with mental health conditions experience relevant disparities in cancer care, including lower access to screening and a higher likelihood of late-stage or emergency diagnoses.

**WHAT THIS STUDY ADDS**

- This is the first Italian population-based study to systematically assess diagnostic pathways and short-term outcomes for colon cancer in individuals with pre-existing mental health conditions using linked cancer registry and healthcare data. The study shows that mental health conditions, particularly dementia, substance use and anxiety-related conditions, are associated with higher odds of emergency diagnosis and lower odds of screening detection, independent of comorbidities and sociodemographic factors.

**HOW THIS STUDY MIGHT AFFECT RESEARCH, PRACTICE OR POLICY**

- These findings provide robust evidence to inform cancer policy and service design, highlighting the need for targeted strategies to reduce diagnostic delays in individuals with mental health conditions. Integrating mental health and cancer care, enhancing screening accessibility and prioritising early detection in vulnerable subgroups could help reduce avoidable mortality and address long-standing inequalities in cancer outcomes.

## Introduction

 In Italy, similar to other European countries, more than one in five individuals experiences at least one mental health conditions, with anxiety and depression being the most prevalent conditions.^1^ Individuals with mental health conditions have a lower life expectancy of up to 20 years compared with the general population, predominantly due to physical diseases,^2^ disparities in healthcare access and quality of care.^3^ While some people with mental health conditions may be at higher risk of developing cancer due to tobacco smoking and other risk factors, they are also more likely to face disparities in screening, diagnosis and treatment of cancer.^4^ In the case of colon cancer, one of the leading causes of cancer-related mortality globally, a recent UK study^5^ found that individuals with mental health conditions presenting with red-flag colon cancer symptoms were 28% less likely to undergo timely investigation with colonoscopy compared with those without. These individuals were also more frequently diagnosed through emergency pathways, a diagnostic route associated with advanced disease stages and poorer prognosis.^6 7^

Evidence on cancer diagnostic pathways in individuals with mental health conditions is scarce, with no population-based studies in Europe. A single Danish study examined these pathways in patients with pre-existing psychiatric conditions but only addressed cases initiated in primary care or diagnosed through unplanned hospital admissions.^8^

A greater understanding of the physical healthcare pathways experienced by people with mental health conditions, particularly in relation to emergency and advanced-stage cancer diagnoses, is essential for designing effective strategies for earlier detection and improves health outcomes. This study aims to examine variations in diagnostic routes, stage at cancer diagnosis and short-term mortality by pre-existing mental health conditions, among patients with colon cancer diagnosed in northern Italy, accounting for their sociodemographic characteristics and physical comorbidities.

## Methods

### Study design and study population

A retrospective, population-based cohort study was conducted examining data from the Milan Cancer Registry, linked to administrative health databases from the Agency for Health Protection of Milan (ATS Milan), which is part of the Lombardy Healthcare System, northern Italy. The study area includes the provinces of Milan and Lodi, comprising approximately 3.5 million residents. Deterministic record linkage was performed using anonymised individual health-system beneficiary codes. The cohort included colon cancer cases (ICD-O-3 topographic codes C18-C19 and behaviour code 3) diagnosed in 2014–2020 among residents of the ATS Milan area. The study followed the Strengthening the Reporting of Observational Studies in Epidemiology reporting guidelines.^9^

### Data sources and variable definitions

Data on gender, age, cancer site, date of diagnosis and stage at diagnosis were obtained from the Milan Cancer Registry, which is part of the Italian Association of Cancer Registry (AIRTum) and the International Association of Cancer Registries.

Patients’ socioeconomic level has been defined using the deprivation index calculated from Italian census data at the census section level, adopting the Rosano revised version of the Caranci index.^10^ The index considers five socioeconomic traits of the resident population and is divided into five levels corresponding to the quintiles of its distribution (1–5, 1 indicating the least deprived level and 5 the most deprived one).

Physical comorbidities were defined using the total number of physical conditions per subject, according to the criteria and codes established by the 2017 Lombardy Region’s deliberation n° X/6164, linking health records up to 6 months precancer diagnosis. Five non-mutually exclusive chronic condition categories were considered: type 2 diabetes and cardiovascular, neurological, genitourinary and cerebrovascular diseases.

We used a validated algorithm^11^ developed by ATS Milan for classifying individuals according to different types of mental conditions or the absence of any mental conditions. This algorithm integrates data from multiple routinely collected administrative health data, including outpatient and inpatient records, pharmaceutical prescriptions and data from emergency departments, community psychiatric care and residential and home-based social-health services. Data from disability registries, pathology-specific exemptions from copayment of healthcare costs, the Mortality Registry (ReNCaM) and the Chronic Disease Patient Database (BDA Chronicity) were included. The classification of specific MI diagnoses was based on the International Classification of Diseases, 10th Revision, using codes F00–F99 (online supplemental table 1).

For the analyses, categories F1, F5 and F6 were combined into a single group due to similarities in diagnostic features or psychopathological mechanisms. Additionally, only categories with at least 100 subjects were included in the analyses to ensure sufficient statistical power and robust estimates.

### Definition of routes to diagnosis

‘Route to diagnosis’ (RtD) refers to the sequence of interactions between a patient and the healthcare system, leading to a cancer diagnosis.^12^ We developed an algorithm to infer the RtD for each study subject based on healthcare contacts in the 6 months preceding cancer diagnosis, in line with previous UK^12^ and international studies.^7^ Details of the algorithm development have been previously described.^13^ RtD were classified into three mutually exclusive categories: (1) screening, (2) emergency presentation (EP) and (3) inpatient/outpatient visits (IP/OP).

### Statistical analysis

We examined sociodemographic characteristics, number of physical comorbidities, stage at diagnosis and short-term mortality for patients with different types of mental health conditions and for those with no mental health conditions. Comparisons between groups were conducted using χ^2^ tests. Logistic regression models were fitted to estimate crude ORs (data not shown) and adjusted ORs (aORs) of experiencing emergency versus non-emergency routes to diagnosis. Additionally, a multinomial regression model was fitted to estimate the ORs of emergency or screening route versus the inpatient/outpatient route as the reference group.

Short-term mortality (30 days, 6 months and 1 year) was also evaluated with logistic regression models.

The primary explanatory variable was the presence of at least one mental health condition. Additional models included the total number of mental health conditions (1 or ≥2) or specific diagnoses of mental health conditions (dementia and related cognitive conditions, substance use/behavioural syndromes/personality-related conditions, depression/mood-related conditions and anxiety) as explanatory variables. As potential confounders, the number of physical comorbidities (0, 1, 2, or ≥3), sociodemographic characteristics (age, gender, deprivation index, marital status, employment status, education level) and cancer stage at diagnosis were considered.

Statistical significance was defined as p value <0.05. We used SAS software (V.9.4, SAS Institute, Cary, North Carolina) for data managing and all statistical analyses.

## Results

### Participant characteristics

The study included 11 676 patients with colon cancer (median age: 73.0 years, IQR): 66–82 years; 47.3% women). Of these, 1895 (16.2%) had a pre-existing mental health condition. Individuals with mental health conditions, compared with those without, were older, more frequently widowed, with lower educational levels and were more frequently diagnosed with colon cancer following EP (43.8% vs 33.8%) and less frequently via screening (4.6% vs 9.1%) (table 1).

**Table 1:** Characteristics of colon cancer patients with and without pre-existing mental health conditions

(*%column*)	Total	Mental health condition		P value
		NO	Any	
	N=11676100%	N=9.78183.8%	N=1895160.2%	
Age				<0.001
* *<50	527	468	59	
	4.5%	4.8%	3.1%	
50–59	1.085	949	136	
	9.3%	9.7%	7.2%	
60–69	2250	2002	248	
	19.3%	20.5%	13.1%	
70–79	3912	3318	594	
	33.5%	33.9%	31.4%	
80–89	3405	2686	719	
	29.2%	27.5%	37.9%	
>=90	497	358	139	
	4.3%	3.7%	7.3%	
Sex				0.111
Male	6.148	5.336	812	
	52.7%	54.6%	42.9%	
Female	5.528	4.445	1.083	
	47.3%	45.5%	57.2%	
Deprivation index				
1	2471	2089	382	0.079
	21.6%	21.8%	20.6%	
2	2015	1717	298	
	17.6%	18.0%	16.1%	
3	1901	1592	309	
	16.6%	16.6%	16.6%	
4	2073	1725	348	
	18.2%	18.0%	18.7%	
5	2964	2444	520	
	26.0%	25.6%	28.0%	
Marital status				
Single	1226	1013	213	<0.001
	10.6%	10.5%	11.3%	
Married	6928	6007	921	
	59.8%	62.0%	48.7%	
Widowed	2625	1984	641	
	22.7%	20.5%	33.9%	
Divorced	380	310	70	
	3.3%	3.2%	3.7%	
Educational level				<0.001
None/primary school	3121	2500	621	
	26.7%	25.6%	32.8%	
Secondary school	3402	2858	544	
	29.1%	29.2%	28.7%	
Diploma/degree/PhD	3885	3281	604	
	33.3%	33.5%	31.9%	
Route to diagnosis				<0.001
Emergency presentation (EP)	4.054	3.236	818	
	35.5%	33.8%	43.8%	
Screening	951	866	85	
	8.3%	9.1%	4.6%	
IP/OP	6.424	5.459	965	
	56.2%	57.1%	51.7%	
Stage at diagnosis				0.196
1–2	4872	4129	743	
	45.1%	45.4%	43.7%	
3–4	5934	4975	959	
	54.9%	54.7%	56.4%	

IP/OP, inpatient/outpatient visits.

Absolute value totals may not sum to 100% due to missing data. Percentages are calculated for each variable based on the total number of non-missing values. Missing data for each variable: deprivation index 2.2%, marital status 4.4%, educational level 10.9%, diagnostic pathway 2.1%, stage at diagnosis 7.5%.

The most frequent pre-existing mental health conditions among patients with colon cancer included diagnoses of depression/mood-related conditions (F3) (9.6%), dementia and related cognitive conditions (F0) (4.6%) and anxiety (F4) (3.3%). Moreover, diagnoses of substance use/behavioural syndromes/personality-related conditions (F1, 5, 6) were experienced by 1.1% of patients, 0.8% had schizophrenia (F2) and 0.5% had learning and intellectual disability (F7), development-related conditions (F8) or childhood and adolescence-related conditions (F9). Data for the most common mental health conditions are shown in table 2. Most patients with dementia and related cognitive conditions were aged 80 years or over (65.4%), whereas other mental conditions also affected younger age groups. Indeed, the median age and IQR varied across diagnoses: 82 years (IQR: 78–87) for dementia and related cognitive conditions, 70 years (IQR: 59–78) for substance use/behavioural syndromes/personality-related conditions, 71 years (IQR: 61–77.5) for schizophrenia, 78 years (IQR: 71–83) for depression/mood-related conditions, 76 years (IQR: 65–82) for anxiety and 68.5 years (IQR: 56–81) for intellectual disability, developmental-related conditions, childhood and adolescence-related conditions or autism. While substance use/behavioural syndromes/personality-related conditions were more common among men, other mental conditions affected women more frequently.

**Table 2:** Patients’ characteristics by specific mental health condition

(*% column*)	Dementia and related cognitive conditions	Substance use/behavioural syndromes/personality-related conditions	Depression/mood-related conditions	Anxiety-related conditions
	Dementia and related organic mental disorders (including Alzheimer’s disease, vascular and secondary dementias, non-substance-induced amnesic and delirium syndromes, and other mental or behavioural disorders due to brain damage or dysfunction).	Mental and behavioural disorders related to substance use, eating and sleep disturbances, sexual and gender identity conditions, personality and impulse control disorders, puerperal and psychosocial-related disorders, and other non-organic or unspecified behavioural syndromes	Mood disorders, including manic and depressive episodes, bipolar disorder, recurrent and persistent depressive disorders, and other or unspecified affective conditions	Neurotic, anxiety, and stress-related disorders, including phobic and other anxiety disorders, OCD, reactions to severe stress, dissociative and somatoform disorders.
	n=53740.6%	n=13310.1%	n=112490.6%	n=39030.3%
Age				
*<50*	7	10	25	19
	1.3%	7.5%	2.2%	4.9%
*50–59*	11	25	72	49
	2.1%	18.8%	6.4%	12.6%
*60–69*	27	30	157	64
	5.0%	22.6%	14.0%	16.4%
*70–79*	141	44	390	127
	26.3%	33.1%	34.7%	32.6%
*80–89*	274	21	415	116
	51.0%	15.8%	36.9%	29.7%
*≥90*	77	3	65	15
	14.3%	2.3%	5.8%	3.9%
Sex				
Male	224	81	440	169
	41.7%	60.9%	39.2%	43.3%
* *Female	313	52	684	221
	58.3%	39.1%	60.9%	56.7%
Deprivation index				
*1–2*	187	43	434	131
	35.5%	32.8%	39.3%	34.4%
*3–5*	340	88	670	250
	64.5%	67.2%	60.7%	65.6%
Educational level				
None/primary school/secondary school	391	85	658	224
	72.8%	66.9%	63.0%	61.5%
Diploma/degree/PhD	128	42	387	140
	24.7%	33.1%	37.0%	38.5%
Diagnostic pathway				
Emergency presentation (EP)	285	72	434	181
	53.9%	54.5%	39.3%	46.7%
Screening	4	6	61	25
	0.8%	4.5%	5.5%	6.4%
IP/OP	240	54	610	182
	45.4%	40.9%	55.2%	46.9%
Stage at diagnosis				
* 1–2*	197	54	465	148
	44.8%	45.0%	44.9%	41.9%
*3–4*	243	66	571	205
	55.2%	55.0%	55.1%	58.1%

IP/OP, inpatient/outpatient visits; OCD, obsessive-compulsive disorder.

Among patients with dementia and related cognitive conditions or substance use/behavioural syndromes, more than one-in-two (53.9% and 54.5%, respectively) were diagnosed with cancer following an EP, and only a tiny minority via screening (0.8% and 4.5%, respectively). Out of all patients with a mental health condition, those affected by depression and related cognitive conditions had the lowest proportion of EP (39.3%). On the other hand, while patients with anxiety had the highest frequency of screening-detected cancers (6.4%), it was still lower compared with patients without mental health condition (9.1%).

Absolute value totals may not sum to 100% due to missing data. Percentages are calculated for each variable based on the total number of non-missing values. Missing data for each variable: deprivation index 6.5%, educational level 5.9%, diagnostic pathway 1.4%, stage at diagnosis 10.8%.

### Multivariable analyses for the likelihood of EP by mental health diagnoses and patient characteristics

Multivariable logistic regression (table 3) showed a significantly higher likelihood of cancer diagnosis following EP for patients with dementia and related cognitive conditions (aOR 1.69, 95% CI 1.41 to 2.03), substance use/behavioural syndromes/personality-related conditions (aOR 1.92, 95% CI 1.34 to 2.75) and anxiety (aOR 1.44, 95% CI 1.16 to 1.79). Moreover, EP was associated with younger and older age groups (<50 years and ≥70 years); with the most deprived; with being single or widowed; and with having at least one physical comorbidity. Furthermore, when the model accounted for having one or at least two mental health conditions, both were significantly associated with EP, with aORs of 1.29 (95% CI 1.15 to 1.44) and 1.70 (95% CI 1.36 to 2.12), respectively (online supplemental table 2).

**Table 3:** Multivariable logistic regression assessing the association between specific mental health conditions, patient sociodemographic characteristics (sex, age, deprivation index, marital status), number of physical comorbidities and emergency presentation (EP) for patients with colon cancer

	EP adjusted OR (95% CI)	P value
Specific mental health conditions (ref. 0)		
Dementia and related cognitive conditions	1.69 (1.41 to 2.03)	<0.001
Substance use/behavioural syndromes/personality-related conditions	1.92 (1.34 to 2.75)	<0.001
Depression/mood-related conditions	0.99 (0.87 to 1.13)	0.887
Anxiety-related conditions	1.44 (1.16 to 1.79)	<0.001
Sex (Ref. M)		
F	0.85 (0.78 to 0.92)	<0.001
Age (ref. 60–69)		
<50	2.63 (2.14 to 3.23)	<0.001
50–59	1.20 (1.01 to 1.42)	0.038
70–79	1.46 (1.30 to 1.65)	<0.001
≥80	2.05 (1.81 to 2.32)	<0.001
Deprivation index (ref. 1)		
2	1.09 (0.96 to 1.24)	0.191
3	1.11 (0.98 to 1.27)	0.113
4	1.17 (1.03 to 1.32)	0.017
5	1.33 (1.19 to 1.49)	<0.001
Marital status (ref. married)		
Single	1.29 (1.13 to 1.47)	0.001
Widowed	1.32 (1.18 to 1.47)	<0.001
Divorced	1.12 (0.90 to 1.41)	0.314
Physical comorbidities count (ref. 0)		
1	1.11 (1.01 to 1.22)	0.031
2	1.22 (1.08 to 1.39)	0.002
3+	1.59 (1.33 to 1.90)	<0.001

The multivariable logistic regression model was adjusted for gender, age, deprivation index, marital status and physical comorbidities count.

Multinomial logistic regression examining the odds of cancer being diagnosed following EP or screening versus the inpatient/outpatient route revealed on the one hand increased odds of EP for people with mental health conditions compared with those without (aOR 1.32, 95% CI 1.19 to 1.47) and on the other hand reduced odds of screening-detected cancer (aOR 0.78, 95% CI 0.60 to 0.99) (online supplemental figure 1). Reduced odds of cancer detection via screening were observed in patients who were single, widowed or divorced compared with their married counterparts as well as in those with at least one physical comorbidity. When specific mental health categories were analysed, patients with dementia and related cognitive conditions had significantly lower odds of screening-detected colon cancer compared with subjects without (aOR 0.27, 95% CI 0.08 to 0.86); for other conditions, the odds of screening detection were also lower but did not reach statistical significance.

### Short-term mortality

Patients with mental health conditions had significantly higher mortality at 30 days (aOR 1.36, 95% CI 1.09 to 1.70), 6 months (aOR 1.32, 95% CI 1.14 to 1.54) and 1 year (aOR 1.47, 95% CI 1.29 to 1.68) compared with those without, independently of physical chronic conditions and patients’ characteristics. Similarly, patients diagnosed through EP exhibited significantly higher mortality at these time points compared with those diagnosed via the IP/OP route. Mortality increased with an increasing number of physical comorbidities, with an aOR of 2.14 (95% CI 1.72 to 2.67) for 1-year mortality in patients with three or more versus no comorbidities, independently of patient characteristics and stage at diagnosis (table 4). In the model examining specific mental health categories, patients with dementia and related cognitive conditions showed a higher mortality at 30 days (aOR 1.73, 95% CI 1.24 to 2.40), 6 months (aOR 1.76, 95% CI 1.38 to 2.24) and 1 year (aOR 1.84, 95% CI 1.46 to 2.32) (online supplemental table 3). A similar trend was observed for other conditions but did not reach statistical significance.

**Table 4:** Short-term mortality in colon cancer: multivariable logistic regression

	30-day mortality adjusted OR (95% CI)	P value	<6 months mortality adjusted OR (95% CI)	P value	1-year mortality adjusted OR (95% CI)	P value
Mental health condition (ref. NO)						
Any	1.36 (1.09 to 1.70)	0.008	1.32 (1.14 to 1.54)	<0.001	1.47 (1.29 to 1.68)	<0.001
Diagnostic pathway (ref. IP/OP)						
Screening	0.09 (0.01 to 0.71)	0.021	0.34 (0.20 to 0.56)	<0.001	0.41 (0.29 to 0.59)	<0.001
Emergency presentation (EP*)*	3.15 (2.57 to 3.85)	<0.001	2.14 (1.89 to 2.41)	<0.001	2.01 (1.81 to 2.24)	<0.001
Sex (ref. M)						
F	0.86 (0.69 to 1.06)	0.147	0.77 (0.68 to 0.88)	<0.001	0.78 (0.69 to 0.87)	<0.001
Age (ref. 60–69)						
<50	0.55 (0.25 to 1.21)	0.137	0.41 (0.26 to 0.65)	<0.001	0.45 (0.31 to 0.65)	<0.001
50–59	0.92 (0.52 to 1.61)	0.758	0.70 (0.51 to 0.96)	0.027	0.76 (0.59 to 0.99)	0.041
70–79	1.47 (1.02 to 2.13)	0.040	1.54 (1.26 to 1.88)	<0.001	1.39 (1.18 to 1.65)	<0.001
≥80	3.38 (2.37 to 4.82)	<0.001	2.84 (2.32 to 3.46)	<0.001	2.73 (2.30 to 3.24)	<0.001
Deprivation index (ref. 1)						
2	0.86 (0.63 to 1.17)	0.324	0.96 (0.79 to 1.17)	0.687	0.87 (0.73 to 1.03)	0.104
3	0.91 (0.67 to 1.25)	0.557	1.04 (0.85 to 1.27)	0.696	1.03 (0.86 to 1.22)	0.759
4	1.06 (0.79 to 1.42)	0.695	1.05 (0.87 to 1.27)	0.598	1.05 (0.88 to 1.24)	0.591
5	0.84 (0.64 to 1.11)	0.221	1.08 (0.91 to 1.28)	0.394	1.03 (0.88 to 1.20)	0.710
Physical comorbidities count (ref. 0)						
1	1.10 (0.87 to 1.38)	0.431	1.22 (1.06 to 1.41)	0.005	1.29 (1.14 to 1.46)	<0.001
2	1.51 (1.14 to 1.99)	0.004	1.55 (1.29 to 1.86)	<0.001	1.61 (1.37 to 1.90)	<0.001
3+	1.90 (1.34 to 2.68)	<0.001	2.02 (1.59 to 2.55)	<0.001	2.14 (1.72 to 2.67)	<0.001
Stage at diagnosis (ref. 1)						
2	2.07 (1.24 to 3.48)	0.006	1.34 (1.00 to 1.78)	0.050	1.32 (1.04 to 1.69)	0.028
3+	5.43 (3.35 to 8.80)	<0.001	6.36 (4.91 to 8.23)	<0.001	6.95 (5.58 to 8.64)	<0.001
Marital status (ref. married)						
Single	1.68 (1.22 to 2.31)	0.002	1.62 (1.32 to 1.99)	<0.001	1.45 (1.21 to 1.75)	<0.001
Widowed	1.26 (0.99 to 1.61)	0.064	1.42 (1.22 to 1.65)	<0.001	1.38 (1.20 to 1.59)	<0.001
Divorced	1.29 (0.73 to 2.28)	0.387	1.09 (0.77 to 1.56)	0.619	1.03 (0.75 to 1.41)	0.870

The multivariable logistic regression model assessing short-term mortality in colon cancer was adjusted for the presence of any mental health condition, diagnostic pathway, sex, age, deprivation index, physical comorbidities count, stage at diagnosis and marital status.

## Discussion

### Summary of key findings

Patients with mental health conditions were significantly less likely to have a screening-detected cancer and more likely to have an emergency cancer diagnosis, with higher mortality, independently of physical comorbidities and sociodemographic characteristics. At least one-in-two patients with dementia and related cognitive conditions, substance use/behavioural/personality-related conditions, or anxiety received a cancer diagnosis following EP. At the same time, this occurred in one-third of individuals with no mental health conditions. This population-based evidence highlights the need for appropriate support and improved pathways for vulnerable patients, to reduce disparities in cancer diagnosis and to improve health outcomes.

### Comparison with the existing literature

Despite the increasing multimorbidity burden among Western populations, with mental health conditions and cancer being among the most common conditions, there is limited research exploring the interplay between them and the impact on diagnostic pathways. To our knowledge, this is one of the few studies providing population-level evidence, utilising linked electronic health records, on routes to cancer diagnosis by mental health conditions in Europe. Studies on emergency cancer diagnosis in this population have previously been conducted in New Zealand^14^ and the USA.^15 16^

The lower likelihood of screening-detected cancer and increased risk of EP among individuals with mental health conditions found in our study is consistent with international evidence documenting systemic barriers to healthcare in psychiatric patients.^8 17 18^

Despite the availability of population-wide screening programmes for colorectal cancer, the most common RtD is following symptomatic presentation,^19^ accounting for 85%–90% of all colorectal cancer diagnoses in the UK. Individuals with mental health conditions may experience difficulties in identifying new symptoms, interpreting them as potential indicators of cancer, appreciating their clinical significance^20^ and communicating them effectively. Although insufficient or delayed help-seeking for cancer symptoms is likely a significant contributor to EPs, limited research has specifically addressed this issue in individuals with mental health conditions.

It is evident that, in addition to cognitive and systemic barriers, psychological and experiential factors may also act as deterrents for individuals with mental conditions when it comes to engaging with cancer screening or diagnostic services. There is evidence to suggest that individuals with lived experiences of physical or sexual abuse, particularly during childhood or in institutional settings, may evade intimate or invasive procedures due to trauma-related distress or fear of retraumatisation.^21^ Furthermore, prior experiences of coercive treatment within the mental healthcare system have been demonstrated to generate long-lasting mistrust in healthcare providers, thereby further complicating engagement with preventive care.^22^ The invasive nature of some diagnostic procedures, such as colonoscopies, can act as a specific deterrent in these populations, highlighting the need for trauma-informed approaches to screening. Moreover, competing demands may particularly affect people with mental health conditions, where the care needs related to their mental health conditions dominate the attention of both patients and caregivers, deprioritising cancer-related symptoms.^23^ Symptoms may also be misattributed through the alternative explanation mechanism, where physical symptoms are interpreted as manifestations of mental health conditions. In the context of colon cancer, symptoms such as changes in bowel habits might be attributed by patients or healthcare providers to pre-existing mental health conditions, rather than being recognised as potential indicators of cancer.^24 25^ Recent research in New Zealand has highlighted the prevalence of diagnostic overshadowing and discrimination in primary care among individuals with mental health and substance use conditions. These issues have a detrimental impact on both access to care and perception of the quality of treatment received.^26 27^ To address these issues, targeted strategies are required, including training of clinicians, promoting patient-centred communication and implementing trauma-informed care models.

Moreover, the high burden of physical comorbidities among individuals with mental health conditions compounds these challenges.^28 29^ In our study, patients with at least one comorbidity had significantly higher odds of EP, which was associated with higher mortality, independently of cancer stage at diagnosis.

Fragmented healthcare services, geographical barriers and long waiting times exacerbate disparities in care for patients with mental health conditions.^18^ In systems with general practitioner gatekeeping, cancer diagnoses typically follow primary care presentation. However, severe mental health conditions complicate management in general practice, as limited consultation time poses a significant barrier to addressing both physical and mental health concerns effectively.^30^

This study also emphasises the heterogeneity within mental health condition subcategories, offering a more detailed understanding of how specific conditions may shape diagnostic pathways and clinical outcomes. Patients with dementia and related cognitive conditions are at particularly high risk of EP.

Cognitive decline and memory impairments likely contribute to difficulties in recognising and reporting early symptoms of colon cancer. Patients with dementia and related cognitive conditions are significantly more likely to present with colon cancer when acute complications occur (such as bowel obstruction or perforation) and are also more likely to have cancer diagnosed by chance (ie, incidental discovery) or not until after death.^31^ Except for advanced age, dementia was identified as the most significant individual risk factor for EP in England,^17^ surpassing several other comorbidities and sociodemographic factors that were examined. Additionally, patients with diagnoses of dementia and related cognitive conditions had markedly reduced odds of screening-detected cancer. These conditions likely exacerbate difficulties in navigating preventive healthcare services, warranting targeted interventions to address these gaps. A 2018 meta-analysis reported lower participation in breast, colorectal and cervical cancer screening among older adults with dementia or cognitive impairment compared with those without.^20^ More recent studies have supported these findings, demonstrating significantly lower rates of breast and prostate cancer screening among patients with dementia in the USA^32^ and reduced rates of mammography screening in Taiwan.^33^

Patients with diagnoses of behavioural and personality-related conditions, including those associated with psychoactive substance use, also demonstrated higher odds of EP. Several factors may explain this association. Substance use often disrupts consistent healthcare engagement, with patients tending to prioritise substance-related needs over preventive care.^34^ However, this represents only one dimension of the issue. Mistrust towards the healthcare system, frequently rooted in previous experiences of stigma, discrimination or coercive treatment may further discourage timely healthcare engagement. Additionally, structural barriers such as financial constraints, including the inability to afford transportation or copayments for clinical consultations, can impede access to routine care and early diagnostic services. These overlapping barriers may synergistically contribute to delayed presentation and reduced participation in cancer screening. Previous studies have shown that they are less likely to be screened for cancers.^35^ Differences in cancer survival have been documented for colorectal and breast cancer, with stage at diagnosis and comorbidities identified as key contributors to these disparities. However, the prediagnosis pathways have been investigated by only a limited number of studies.^14^

Patients with diagnoses of anxiety had higher odds of EP, potentially driven by delayed care-seeking behaviour linked to fear of diagnostic procedures or catastrophic interpretations of mild symptoms.^18 36^ Somatisation, a common feature of anxiety, may further obscure early cancer symptoms, delaying diagnosis until complications arise. However, other studies have noted that anxiety conditions are more commonly observed in individuals who participate in cancer screening compared with non-participants. This is partially consistent with our findings, which indicate higher screening rates among individuals with anxiety compared with those with other types of mental health conditions. The association may be partially explained by the heightened health awareness and concern characteristic of health anxiety and hypochondriasis.^37^ This dual role emphasises the complexity of the impact of anxiety on cancer diagnostic pathways and outcomes.

Our study revealed significantly higher mortality among patients with mental health conditions at 30 days, 6 months and 1 year. While it is well established that patients with mental health conditions are more likely to be exposed to additional risk factors such as smoking, substance use and obesity,^38 39^ our findings indicate that the elevated mortality persisted even after adjusting for the comorbidity burden.

### Implications for public health

Our findings highlighted substantial disparities in cancer diagnostic pathways and health outcomes among individuals with mental health conditions, underscoring an urgent need for public health strategies to address these inequities. A greater integration between mental health and cancer prevention and care services could improve health outcomes for vulnerable populations. Public health policies should prioritise outreach, awareness campaigns and care coordination to reduce diagnostic delays and promote equity in cancer care. Additionally, training for healthcare professionals could improve cancer communication as well as cancer-symptom recognition, reducing the risk of diagnostic overshadowing, when caring for patients with mental health conditions.

In the context of public health research and practice, the use of inclusive and non-stigmatising language is essential to avoid reinforcing discrimination towards individuals with mental health conditions or problematic substance use. For example, referring to ‘mental health conditions’ rather than ‘mental disorders’, is in line with a more person-centred and respectful approach. Adopting respectful terminology contributes to reducing stigma and promoting equity in health communication and care delivery.

### Strengths and limitations

We developed a robust methodology to classify cancer diagnosis pathways using linked cancer registry and administrative health data. This approach leverages routinely collected, large-scale datasets to enhance cancer surveillance and identify areas for improvement. The use of high-quality cancer registry data underpins the reliability and validity of our findings, while the application of a validated algorithm for identifying mental health conditions ensures accurate classification of psychiatric conditions. Moreover, the inclusion of specific mental health categories provides nuanced insights into their differential impact on cancer care pathways and outcomes.

However, this study is not without limitations. The absence of detailed clinical data may have resulted in the underestimation of psychiatric conditions due to potential underdiagnosis or misclassification, especially for conditions that are less severe or affect marginalised groups, as people who experience them are less likely to access healthcare. Due to the limited sample size, the analysis relies on broad mental health categories rather than specific ones, which may limit the specificity of the findings. Missing data on cancer stage at diagnosis could influence risk estimates, although the observed associations suggest minimal bias.

## Conclusions

Using population-based data has shown that individuals with mental health conditions had a higher likelihood of colon cancer diagnosed after EP and higher mortality compared with those without mental health conditions, independently of physical conditions and other patient characteristics. Addressing these disparities requires a healthcare system prioritising equitable access to timely, effective and non-discriminatory care for vulnerable groups. Our findings underscore the critical need for tailored interventions supporting access to screening and early cancer diagnosis for individuals with mental health conditions to improve survival. Achieving this goal will necessitate comprehensive improvements across the cancer care continuum, including enhanced and inclusive cancer screening programmes, strengthened primary care and diagnostic services, streamlined referral pathways and robust monitoring of outcomes for individuals with mental health conditions.

## Supplementary material

10.1136/bmjment-2025-301733 online supplemental file 1

## Data Availability

Data are available upon reasonable request.
