# Supplementary material for: Emergency colon cancer diagnosis in people with mental health conditions: a population-based cohort study in northern Italy
Source: BMJ Ment Health. 2025 Jul 1;28(1):e301733. doi: 10.1136/bmjment-2025-301733 (PMC12215114; doi:10.1136/bmjment-2025-301733)
Supplement: online supplemental file 1 [file bmjment-28-1-s001.docx]

**Supplementary materials**

**Supplemental Table 1. Mental health categories according to ICD-10 classification.**

| **ICD-10-Short description** | **Description based on ICD-10 classification** | **Included conditions** |
| --- | --- | --- |
| F0- Dementia and related cognitive conditions | Organic, including symptomatic, mental disorders | Dementia in Alzheimer disease; vascular dementia; dementia in other diseases classified elsewhere; organic amnesic syndrome, not induced by alcohol and other psychoactive substances; delirium, not induced by alcohol and other psychoactive substances; other mental disorders, personality and behavioural disorders due to brain damage, dysfunction and physical diseases; unspecified organic or symptomatic mental disorder. |
| F1-Substance use | Mental and behavioural disorders due to psychoactive substance use | Mental and behavioural disorders due to use of alcohol, opioids, cannabinoids, sedatives or hypnotics, cocaine, other stimulants, including caffeine, hallucinogens, tobacco, volatile solvents, multiple drug and other psychoactive substances. |
| F2-Schizophrenia | Schizophrenia, schizotypal and delusional  disorders | Schizophrenia; schizotypal disorder; persistent delusional disorders; acute and transient psychotic disorders; Induced delusional disorder; schizoaffective disorders; other nonorganic psychotic disorders; unspecified nonorganic psychosis. |
| F3-Depression/Mood- related conditions | Mood [affective] disorders | Manic episode; bipolar affective disorder; depressive episode; recurrent depressive disorder; persistent mood [affective] disorders; other mood [affective] disorders; unspecified mood [affective] disorder. |
| F4-Anxiety- related conditions | Neurotic, stress-related and somatoform  disorders | Phobic anxiety disorders; other anxiety disorders; obsessive-compulsive disorders; reaction to severe stress, and adjustment disorders; dissociative [conversion] disorders; somatoform disorders; other neurotic disorders. |
| F5-Behavioral syndromes | Behavioural syndromes associated with physiological disturbances and physical factors | Eating disorders; nonorganic sleep disorders; sexual dysfunction, not caused by organic disorder or disease; mental and behavioural disorders associated with the puerperium, not elsewhere classified; psychological and behavioural factors associated with disorders or diseases classified elsewhere; abuse of non-dependence-producing substances; unspecified behavioural syndromes associated with physiological disturbances and physical factors. |
| F6-Personality- related conditions | Disorders of adult personality and behaviour | Specific personality disorders; mixed and other personality disorders; enduring personality changes, not attributable to brain damage and disease; habit and impulse disorders; gender identity disorders; disorders of sexual preference psychological and behavioural disorders associated with sexual development and orientation; other disorders of adult personality and behaviour; unspecified disorder of adult personality and behaviour. |
| F7- Learning and intellectual disability | Mental retardation | Mild, moderate, severe, profound, other and unspecified mental retardation. |
| F8-Development- related conditions | Disorders of psychological development | Specific developmental disorders of speech and language, scholastic skills, motor function;mixed specific developmental disorders; pervasive developmental disorders; other disorders of psychological development unspecified disorder of psychological development. |
| F9-Childhood and adolescence- related conditions | Behavioural and emotional disorders with onset usually occurring in childhood and adolescence | Hyperkinetic disorders; conduct disorders; mixed disorders of conduct and emotions; emotional disorders with onset specific to childhood; disorders of social functioning with onset specific to childhood and adolescence; tic disorders; other behavioural and emotional disorders with onset usually occurring in childhood and adolescence. |

**Supplemental Table 2. Multivariable logistic regression assessing the association between number of mental health conditions patient characteristics, and Emergency presentation (EP) for patients with colon cancer.**

|  | **Emergency presentation adjusted OR (95% IC)** | **P value** |  |
| --- | --- | --- | --- |
|  |  |  |  |
| ***Number of mental health conditions (Ref 0)*** |  |  |  |
| *1* | 1.29 (1.15-1.44) | <0.001 |  |
|  |  |  |  |
| *2+* | 1.70 (1.36-2.12) | <0.001 |  |
|  |  |  |  |
| ***Gender (Ref M)*** |  |  |  |
| *F* | 0.84 (0.77-0.91) | <0.001 |  |
|  |  |  |  |
| ***Age (Ref. 60-69)*** |  |  |  |
| *<50* | 2.65 (2.16-3.25) | <0.001 |  |
|  |  |  |  |
| *50-59* | 1.21 (1.02-1.43) | 0.031 |  |
|  |  |  |  |
| *70-79* | 1.46 (1.30-1.65) | <0.001 |  |
|  |  |  |  |
| *>=80* | 2.06 (1.82-2.33) | <0.001 |  |
|  |  |  |  |
| ***Deprivation index (Ref.1)*** |  |  |  |
| *2* | 1.09 (0.96-1.24) | 0.203 |  |
|  |  |  |  |
| *3* | 1.11 (0.98-1.27) | 0.106 |  |
|  |  |  |  |
| *4* | 1.17 (1.03-1.33) | 0.016 |  |
|  |  |  |  |
| *5* | 1.33 (1.19-1.50) | <0.001 |  |
|  |  |  |  |
| ***Marital status (Ref. Married)*** |  |  |  |
| *Single* | 1.29 (1.12-1.47) | <0.001 |  |
|  |  |  |  |
| *Widowed* | 1.32 (1.19-1.47) | <0.001 |  |
|  |  |  |  |
| *Divorced* | 1.13 (0.90-1.42) | 0.300 |  |
|  |  |  |  |
| ***Physical comorbidities count (Ref 0)*** |  |  |  |
| *1* | 1.11 (1.01-1.22) | 0.032 |  |
|  |  |  |  |
| *2* | 1.23 (1.09-1.40) | 0.001 |  |
|  |  |  |  |
| *3+* | 1.61 (1.35-1.92) | <0.001 |  |
|  |  |  |  |

**Supplemental Table 3.** **Short-term mortality in colon cancer: multivariable logistic regression, considering specific mental health conditions.**

| **Colon Adjusted** | **30 days-mortality (adjusted) OR (95% IC** | **P value** | **<6 months-mortality (adjusted) OR (95% IC)** | **P value** | **one year-mortality (adjusted) OR (95% IC)** | **P value** |  |
| --- | --- | --- | --- | --- | --- | --- | --- |
|  |  |  |  |  |  |  |  |
| **Specific mental health conditions *(Ref. 0)*** |  |  |  |  |  |  |  |
| *Dementia and related cognitive conditions* | 1.73 (1.24-2.40) | 0.002 | 1.76 (1.38-2.24) | <0.001 | 1.84 (1.46-2.32) | <0.001 |  |
| *Substance use/ behavioral syndromes/ personality-related conditions* | 1.41 (0.68-2.95) | 0.357 | 1.14 (0.68-1.92) | 0.616 | 1.38 (0.87-2.18) | 0.171 |  |
|  |  |  |  |  |  |  |  |
| *Depression/ Mood- related conditions* | 0.88 (0.65-1.20) | 0.432 | 0.98 (0.81-1.19) | 0.816 | 1.15 (0.97-1.37) | 0.108 |  |
|  |  |  |  |  |  |  |  |
| *Anxiety-related conditions* | 1.15 (0.72-1.82) | 0.566 | 1.15 (0.85-1.56) | 0.363 | 1.12 (0.85-1.48) | 0.417 |  |
|  |  |  |  |  |  |  |  |
| ***Gender (Ref. M)*** |  |  |  |  |  |  |  |
| *F* | 0.87 (0.71-1.07) | 0.195 | 0.78 (0.68-0.88) | <0.001 | 0.78 (0.70-0.88) | <0.001 |  |
|  |  |  |  |  |  |  |  |
| ***Age (Ref. 60-69)*** |  |  |  |  |  |  |  |
| *<50* | 0.55 (0.25-1.20) | 0.132 | 0.40 (0.25-0.64) | <0.001 | 0.45 (0.31-0.65) | <0.001 |  |
|  |  |  |  |  |  |  |  |
| *50-59* | 0.92 (0.52-1.61) | 0.764 | 0.70 (0.51-0.96) | 0.029 | 0.76 (0.59-0.99) | 0.041 |  |
|  |  |  |  |  |  |  |  |
| *70-79* | 1.48 (1.03-2.15) | 0.036 | 1.54 (1.26-1.88) | <0.001 | 1.39 (1.17-1.65) | <0.001 |  |
|  |  |  |  |  |  |  |  |
| *>=80* | 3.38 (2.36-4.82) | <0.001 | 2.81 (2.30-3.43) | <0.001 | 2.71 (2.28-3.22) | <0.001 |  |
|  |  |  |  |  |  |  |  |
| ***Deprivation index (Ref. 1)*** |  |  |  |  |  |  |  |
| *2* | 0.85 (0.62-1.16) | 0.308 | 0.96 (0.79-1.16) | 0.653 | 0.87 (0.73-1.03) | 0.104 |  |
|  |  |  |  |  |  |  |  |
| *3* | 0.91 (0.66-1.24) | 0.549 | 1.04 (0.85-1.26) | 0.704 | 1.03 (0.86-1.22) | 0.759 |  |
|  |  |  |  |  |  |  |  |
| *4* | 1.06 (0.79-1.43) | 0.678 | 1.05 (0.87-1.27) | 0.595 | 1.05 (0.89-1.24) | 0.591 |  |
|  |  |  |  |  |  |  |  |
| *5* | 0.84 (0.63-1.10) | 0.208 | 1.08 (0.91-1.28) | 0.397 | 1.03 (0.88-1.20) | 0.710 |  |
|  |  |  |  |  |  |  |  |
| ***Physical omorbidities count (Ref. 0)*** |  |  |  |  |  |  |  |
| *1* | 1.10 (0.87-1.39) | 0.413 | 1.23 (1.07-1.41) | 0.005 | 1.29 (1.14-1.46) | <0.001 |  |
|  |  |  |  |  |  |  |  |
| *2* | 1.49 (1.13-1.97) | 0.005 | 1.53 (1.28-1.84) | <0.001 | 1.59 (1.35-1.88) | <0.001 |  |
|  |  |  |  |  |  |  |  |
| *3+* | 1.88 (1.33-2.66) | <0.001 | 2.00 (1.58-2.54) | <0.001 | 2.12 (1.70-2.65) | <0.001 |  |
|  |  |  |  |  |  |  |  |
| ***Stage at diagnosis (Ref. 1)*** |  |  |  |  |  |  |  |
| *2* | 2.07 (1.23-3.47) | 0.006 | 1.33 (1.00-1.78) | 0.052 | 1.32 (1.03-1.68) | 0.028 |  |
|  |  |  |  |  |  |  |  |
| *3+* | 5.45 (3.36-8.83) | <0.001 | 6.39 (4.94-8.28) | <0.001 | 6.98 (5.61-8.69) | <0.001 |  |
|  |  |  |  |  |  |  |  |
| ***Marital status (Ref. Married)*** |  |  |  |  |  |  |  |
| *Single* | 1.68 (1.22-2.31) | 0.002 | 1.62 (1.33-1.99) | <0.001 | 1.46 (1.22-1.76) | <0.001 |  |
|  |  |  |  |  |  |  |  |
| *Widowed* | 1.26 (0.99-1.61) | 0.063 | 1.42 (1.21-1.65) | <0.001 | 1.38 (1.20-1.58) | <0.001 |  |
|  |  |  |  |  |  |  |  |
| *Divorced* | 1.29 (0.73-2.29) | 0.378 | 1.09 (0.77-1.56) | 0.628 | 1.03 (0.75-1.40) | 0.870 |  |
|  |  |  |  |  |  |  |  |

**Supplemental Figure 1. Multinomial regression analysis assessing the association between mental health conditions, patient characteristics, comorbidities, and the diagnostic pathway for colon cancer.**

**
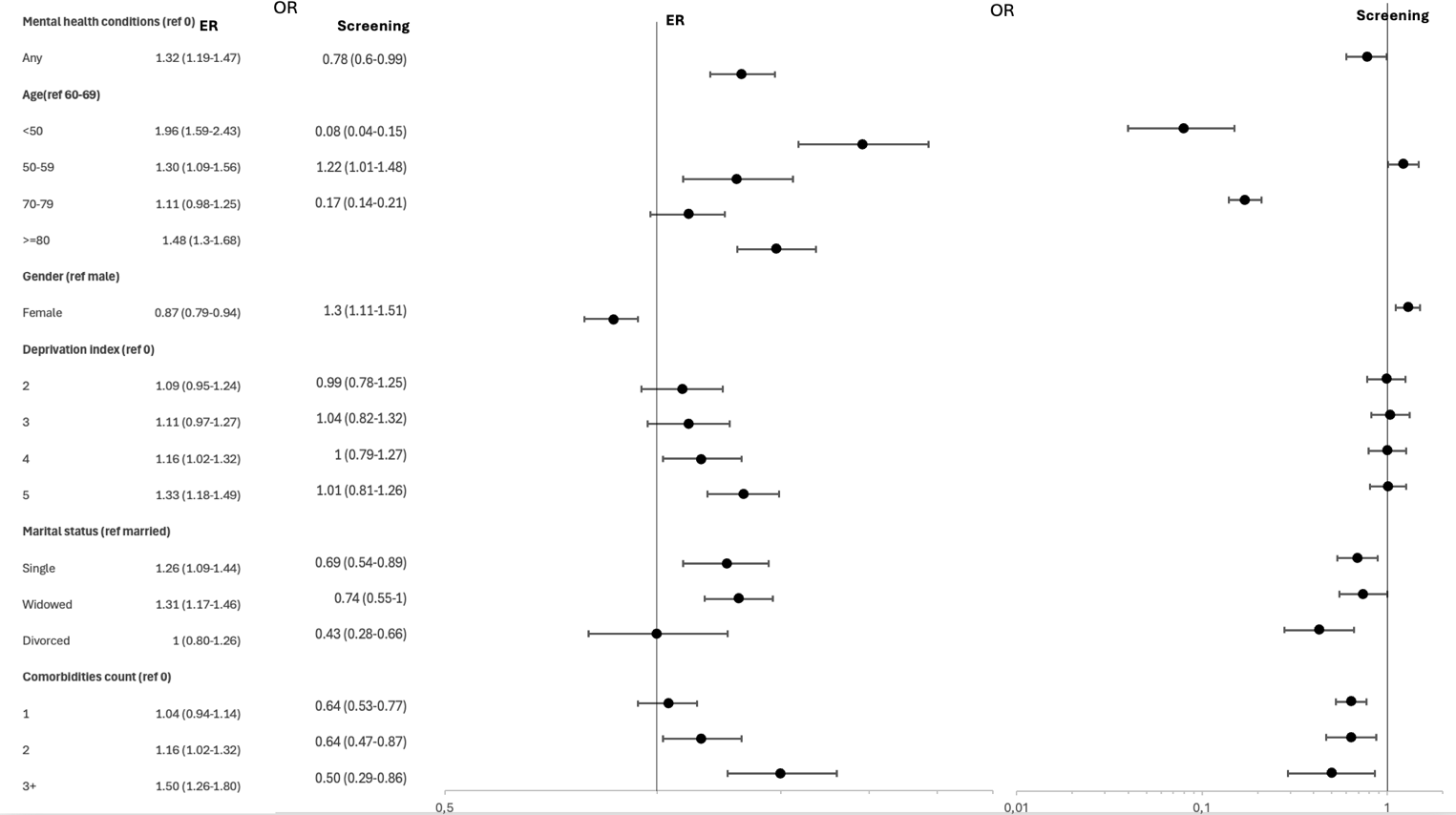
**
